# Aspirin Attenuates Liver Fibrosis via Autophagy Induction

**DOI:** 10.1111/jcmm.70696

**Published:** 2025-07-03

**Authors:** Shenglan Wang, Mengxue Sun, Min Tang, Zixin Yang, Juan Shan, Changqing Yang

**Affiliations:** ^1^ Division of Gastroenterology and Institute of Digestive Disease Tongji Hospital, Tongji University School of Medicine Shanghai China

**Keywords:** aspirin, autophagy, autophagy flux, hepatic fibrosis

## Abstract

The aim of the present study was to explore the effect of the non‐steroidal anti‐inflammatory drugs (NSAID) aspirin on the progression of liver fibrosis and to elucidate its underlying mechanisms, with a specific focus on autophagy. In vitro, the rat hepatic stellate cell line HSC‐T6 was activated using transforming growth factor‐β1 (TGF‐β1). Western blot and real‐time PCR analysis were employed to investigate the effect of aspirin on HSC‐T6 activation and its association with autophagy levels, including key autophagic markers. In vivo study, liver fibrosis was induced in mice via long‐term thioacetamide (TAA) administration. The impact of aspirin on liver fibrosis and function was evaluated using Masson's Trichrome and Sirius Red staining to assess collagen deposition, complemented by serum biochemistry analysis. TGF‐β1 treatment inhibited autophagic flux in activated HSC‐T6 cells, as evidenced by increased LC3II/I and p62 expression. Notably, aspirin effectively attenuated fibrogenesis in these cells, with significantly lower expression levels of α‐SMA and collagen I compared to the TGF‐β1‐treated control group. Concurrently, aspirin restored autophagy flux as indicated by decreased LC3‐II/I and p62 levels, and the effect can be reversed by the autophagy inhibitor chloroquine (CQ). In vivo, aspirin administration markedly attenuated liver fibrosis. Mechanistically, aspirin treatment enhanced autophagic flux, as demonstrated by the accumulation of autolysosomes observed in liver tissues via transmission electron microscopy (TEM). Our study demonstrates that aspirin inhibits liver fibrosis progression by inducing autophagy, highlighting its potential as a therapeutic strategy for liver fibrosis.

**Abbreviations**
ALT: Alanine aminotransferase
AST: Aspartate Transaminase
CQ: chloroquine
ECM: extracellular matrix
GAPDH: glyceraldehyde 3‐phosphate dehydrogenase
GIBCO: Dulbecco's modified Eagle's medium
HCC: hepatocellular carcinoma
HE: Haematoxylin and Eosin
HSCs: hepatic stellate cells
IHC: immunohistochemical
NAFLD: non‐alcoholic fatty liver disease
NSAID: non‐steroidal anti‐inflammatory drugs
ROS: reactive oxygen species
TAA: thioacetamide
TEM: transmission electron microscopy
TGF‐β1: transforming growth factor‐β1
αSMA: Alpha‐Smooth Muscle Actin

## 1 Introduction

Liver fibrosis represents a dysregulated wound‐healing response to chronic liver diseases across diverse etiologies. Characterised by excessive accumulation of extracellular matrix (ECM) proteins, it drives the progression of chronic liver disease toward advanced cirrhosis and, ultimately, hepatocellular carcinoma (HCC) [1]. Importantly, as liver fibrosis and early cirrhosis are dynamic and reversible processes, interventions to regulate hepatic fibrogenesis are critical for preventing cirrhosis and treating liver failure [2]. Therefore, novel therapeutic strategies and drugs for anti‐fibrotic therapy are urgently needed [3].

Previous studies have shown that intrahepatic thrombosis of medium and large vessels is prevalent in cirrhosis patients and represents a key driver of liver fibrosis progression. Platelets have emerged as a promising target for antifibrotic therapeutic development. Consequently, multiple studies examining regular aspirin use have investigated the effect on liver fibrosis [3].

Aspirin, a well‐known anti‐inflammatory drug, functions as a specific inhibitor of cyclooxygenase. By targeting this enzyme, it effectively inhibits the production of prostacyclin, prostaglandins, and thromboxane [4, 5]. As one of the non‐steroidal anti‐inflammatory drugs (NSAIDs), which also include celecoxib and paracetamol, aspirin is widely used for treating pain, inflammation, and fever [6]. In a rat model of cirrhosis induced by thioacetamide (TAA), aspirin treatment led to a significant improvement in liver fibrosis grading compared with controls [7, 8]. These findings suggest that aspirin treatment holds potential as a therapeutic strategy for inhibiting liver fibrosis.

Autophagy is an intracellular degradation pathway that targets cytoplasmic components to lysosomes for degradation [9, 10]. Long‐lived proteins, protein aggregates, and organelles are sequestered by double‐membrane autophagosomes, which then fuse with lysosomes for breakdown. By digesting unwanted or damaged components, recycling materials, and generating energy, autophagy maintains cellular and tissue homeostasis. Its dysfunction has been linked to the development of multiple human diseases [11]. Although accumulating evidence indicates impaired hepatic autophagy in chronic liver diseases [12, 13, 14], the underlying mechanisms remain controversial. Some reports suggest that hyperinsulinemia and downregulation of autophagy pathway proteins (such as autophagy‐related proteins Atg7 and Atg5) are associated with defective autophagosome formation. Conversely, other studies demonstrate impairments in late‐stage autophagosome maturation steps (e.g., vesicle fusion and acidification) rather than autophagosome formation [15, 16, 17]. Given that autophagic dysfunction may regulate liver injury, inflammation, fibrosis, and carcinogenesis [18], elucidating the precise mechanisms governing autophagy is crucial for identifying therapeutic targets in this common and potentially severe liver disorder.

Accumulating evidence suggests that autophagy is crucially involved in inhibiting fibroblast activation and extracellular matrix accumulation during fibrogenesis [19, 20, 21]. In alpha‐1‐antitrypsin (α1AT)‐deficient models, autophagy agonists such as rapamycin reduce mutant α1AT aggregate burden in the liver by enhancing autophagic clearance, thereby attenuating hepatic fibrosis [22]. Recent studies increasingly highlight autophagy as an emerging therapeutic avenue for liver diseases, with its mechanistic links to cellular homeostasis and fibrosis resolution [23]. However, the role of aspirin in regulating autophagy in hepatic stellate cells (HSCs) and its impact on liver fibrosis remain poorly understood.

## 2 Materials and Methods

### 2.1 Cell Culture and Treatment

HSC‐T6 were cultured in Dulbecco's modified Eagle's medium (GIBCO) containing 10% fetal bovine serum (FBS), 1% antibiotics, and maintained in a humidified incubator at 37°C with 5% CO_2_ and 95% air. Aspirin (MCE, HY‐14654) was dissolved in DMSO at a concentration of 10 mM and was stored in a dark bottle at −20°C. The stock was diluted to the required concentration with DMSO when necessary. HSCs were activated by TGF‐β1 (5 ng/mL, Peprotech) and treated with Aspirin for 12 hours. To inhibit autophagy, chloroquine (CQ; 50 nM; Sigma Aldrich) was added to the medium. To induce autophagy, rapamycin (Rapa; 25 ng/mL; Sigma‐Aldrich) was added to the medium.

To inhibit the activity of COX‐1, COX‐2 in vitro, the inhibitors of COX‐1 (sc‐560, 50 μM, MCE), COX‐2 (celecoxib, 50 μM, MCE) were used. The same amount of DMSO was used as a control.

### 2.2 Cell Viability

Cells were seeded in a 96‐well plate at a density of 10,000 cells per well and cultured overnight. HSC‐T6 cells were treated with different concentrations of aspirin (5, 10, 15, 20, 25 mmol/L) (MCE, HY‐14654) for 24 h. Cell Counting Kit‐8 (CCK‐8; Beyotime, China) reagent was added to the cells (10 μL per well). Cells were incubated for 1.5 h, and the viability was measured.

### 2.3 Electron Microscopy

The liver tissue samples were collected and fixed with 3% glutaraldehyde in 0.2 M sodium cacodylate buffered at pH 7.4. Images were acquired using a transmission electron microscope.

### 2.4 Animal Protocol

The animal care and experimental procedures involved in this study were approved by the Institutional Animal Care and Use Committee of Tongji Hospital, Tongji University. All animal procedures were performed under the guidelines established by the Institutional Animal Care and Use Committee of Tongji University College of Medicine and consistent with those established by the National Institutes of Health.

Six‐to‐eight‐week‐old male C57BL/6 mice (purchased from Beijing Vital River Laboratory Animal Technology Co Ltd) were housed in a standard animal facility under a 12‐h light/dark cycle with access to food and water. The mice were randomly assigned to four groups (*n* = 6): Control+PBS, Control+Aspirin, TAA + PBS, TAA + Aspirin. Mice in the TAA groups received intraperitoneal (i.p.) injections of thioacetamide (TAA, Sigma‐Aldrich) at a dose of 0.2 mg/g body weight, administered 3 times weekly for 6 consecutive weeks. Starting from week 3, mice in the aspirin groups were injected intraperitoneally for 4 weeks at a daily dose of 100 mg·kg^‐1^ in the morning, while control groups received an equivalent volume of PBS. Treatments were synchronised across all groups to ensure consistency. At 24 h after the final TAA injection (end of week 6), liver tissues and blood samples were collected. All specimens were immediately stored at −80°C for subsequent biochemical and histopathological analyses.

### 2.5 RNA Isolation and Real‐Time PCR

Total RNA was extracted using TRIzol reagent (Nanjing Jiancheng Bioengineering Institute, Nanjing, China) and treated with RNase‐free DNase (Promega) for 30 min at 37°C. After DNase treatment, RNA was cleaned up using an RNeasy kit (Nanjing Jiancheng Bioengineering Institute, Nanjing, China). RNA was reverse‐transcribed using a first‐strand cDNA kit with random hexamers (Nanjing Jiancheng Bioengineering Institute, Nanjing, China). The expression level of glyceraldehyde 3‐phosphate dehydrogenase (GAPDH) was used as an internal control. The PCR and analysis were performed using the Light Cycler and software (Roche). All primer sequences are synthesised by Huagene (Shanghai, China) and listed in Table S1.

### 2.6 Western Blot Assay

Total protein was extracted from frozen cells and quantified by standard procedures. The protein concentrations were measured by the BCA Protein Assay. The samples were separated on a 10% SDS‐polyacrylamide gel and transferred to a nitrocellulose membrane, which was blocked in 5% skim milk for 1 h and then incubated with primary antibodies overnight at 4°C. After washing, the membranes were incubated with horseradish peroxidase‐labelled secondary antibodies for 1 h at room temperature. A chemiluminescence detection system was used to visualise the bands, and Image J software was used to analyse the density measurement results. All antibodies used in experiments are shown in Table S2.

### 2.7 Masson Staining and Histochemical Stain

Liver specimens were fixed with formalin, embedded in paraffin, sectioned at 4 μm, and processed routinely for H&E staining. Sirius Red and Masson staining (Sigma) were performed on 4 μm‐thick formalin‐fixed paraffin‐embedded tissue sections. Sirius Red and Masson‐stained areas from 10 fields (magnification ×200) from 3 to 6 mice/group were quantified with Image J. Immunohistochemical staining of α‐SMA was performed with a rabbit polyclonal antibody (Abcam). Sections were stained with Sirius Red solution (saturated picric acid containing 0.1% Direct Red 80% and 0.1% Fast Green) to visualise collagen deposition (magnification ×200).

### 2.8 Statistical Analyses

The data were analysed using GraphPad Prism software (Version 9.0, San Diego, CA, USA) and presented as mean ± SEM. Student's t‐test was used to compare the two groups. For multi‐comparisons, analysis of variance (ANOVA) was performed to detect an overall difference among the groups. *p* < 0.05 was considered indicative of statistical significance.

## 3 Results

### 3.1 Aspirin Inhibits Liver Fibrosis Caused by TAA

**FIGURE 1:**
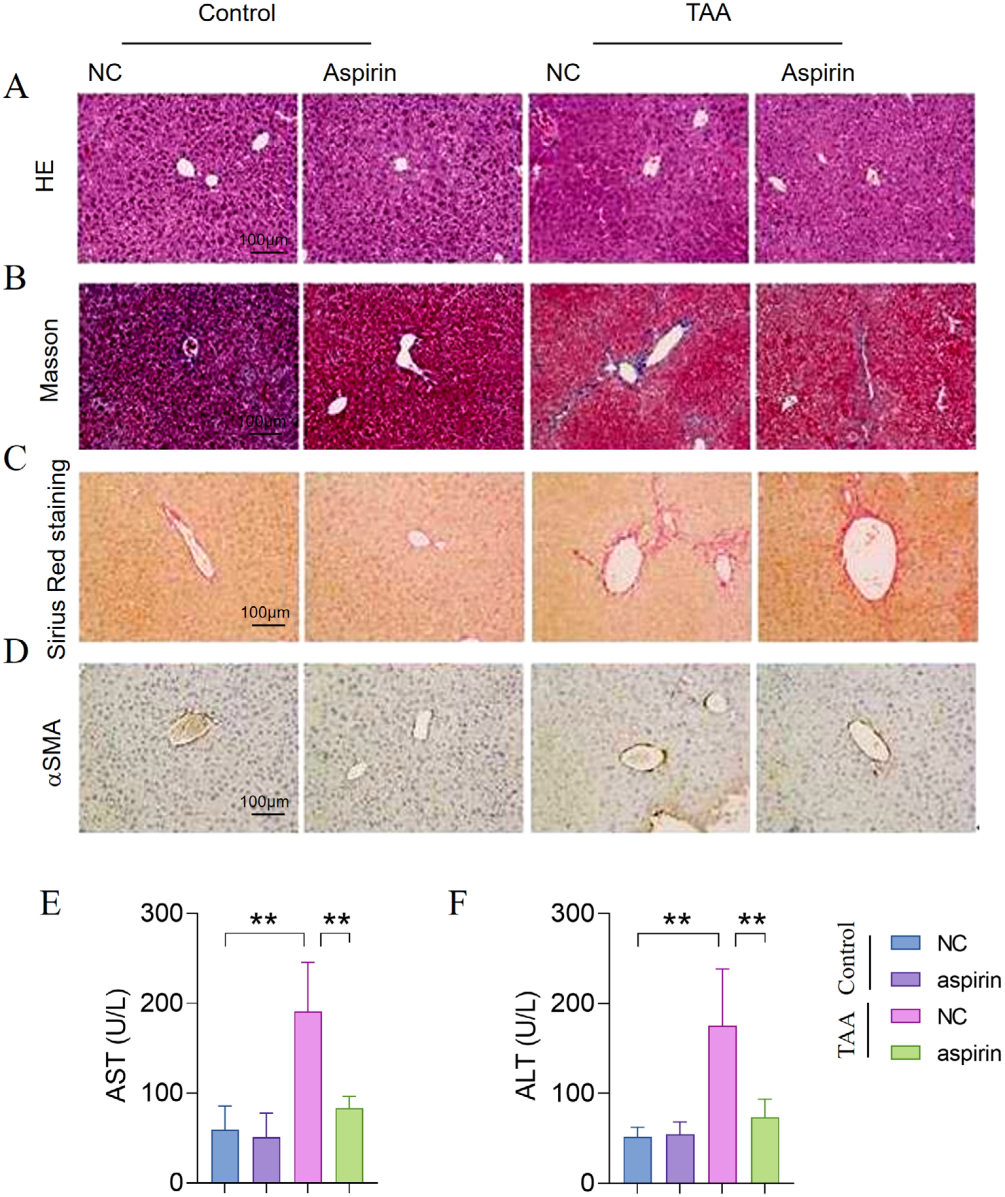
Aspirin inhibits liver fibrosis caused by TAA. (A) HE staining of the indicated group. (B) Masson's trichrome staining of the indicated group. (C) Sirius Red staining of the indicated group. (D) Immunohistochemistry (IHC) staining for αSMA of the indicated group. (E) Serum levels of AST. (F) Serum levels of AST. Statistical comparisons with the TAA group were performed by an unpaired two‐sided t test. **p < 0.01.

To investigate the antifibrotic effects of aspirin, mice were administered aspirin via injection for four consecutive weeks. Haematoxylin and eosin (HE) staining revealed reduced hepatic inflammatory infiltration (Figure 1A), while Masson trichrome and Sirius Red staining of liver sections showed that aspirin significantly inhibited hepatic collagen deposition and fibrosis (Figure 1B,C). Additionally, α‐SMA immunohistochemical (IHC) staining demonstrated that aspirin markedly reduced the activation of HSCls (Figure 1D). Besides, after aspirin treatment, the levels of serum ALT and AST were compared to the control group.

### 3.2 Aspirin Alleviates Hepatic Fibrosis by Promoting Autophagy in Activated HSCs

**FIGURE 2:**
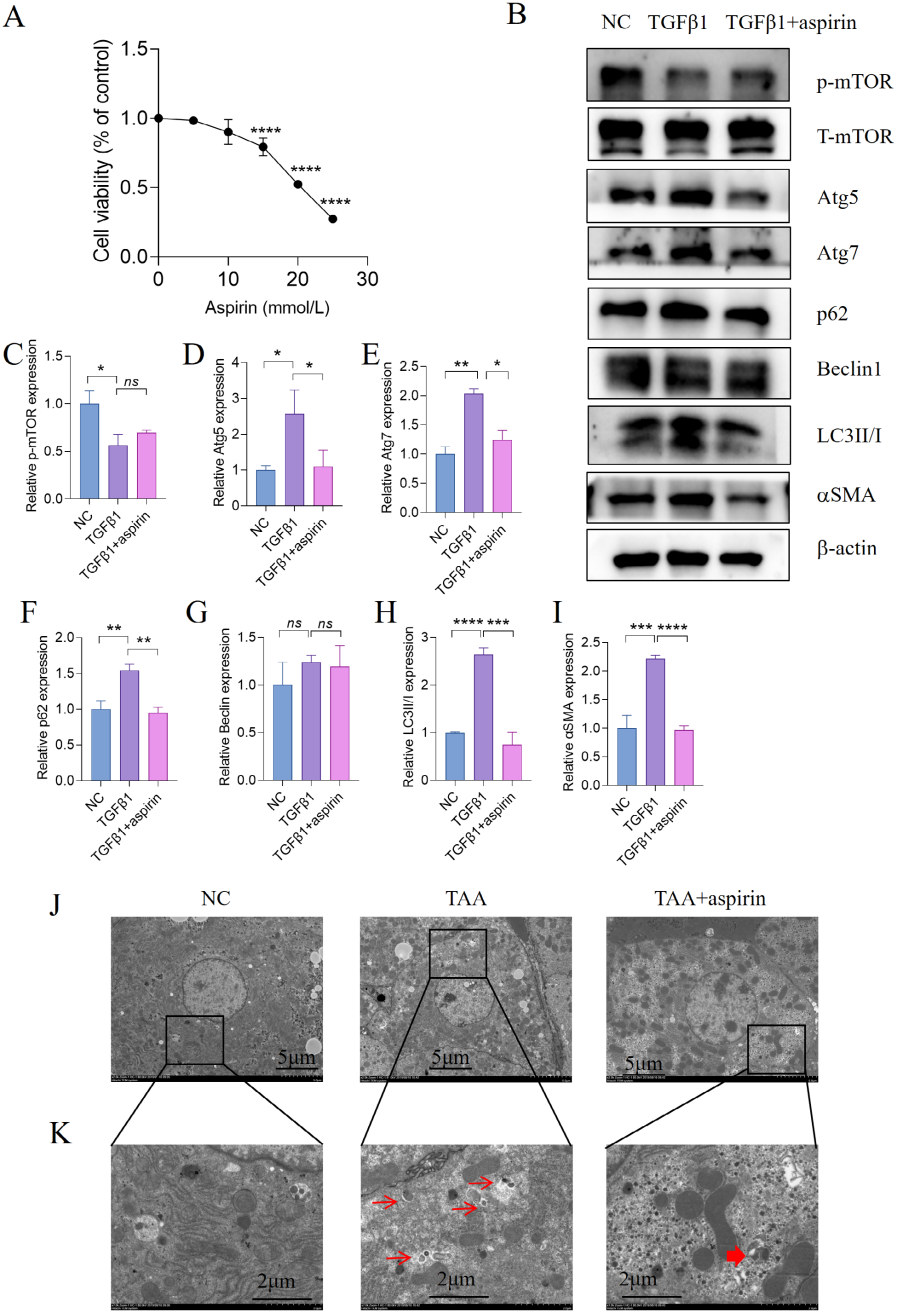
Aspirin alleviates hepatic fibrosis by promoting autophagy in activated HSCs. (A) The viability of HSC‐T6 cells incubated with different concentrations of aspirin for 24 h was measured by the CCK‐8 assay. (B) Protein expression of fibrosis‐related proteins and autophagy‐related proteins in activated HSC‐T6 treated with 10 mmol/L aspirin. (C–I) Quantification of the indicated protein expression normalised to β‐Actin. (J–K) Representative images of TEM observation of the livers of control mice, TAA mice, and TAA mice treated with aspirin, the lower panels are amplified from selected areas in the upper panels. Thick arrows indicate autolysosomes, while thin arrows indicate autophagosomes. Statistical comparisons with the TGFβ1 group were performed by an unpaired two‐sided t test. *p < 0.05, **p < 0.01, ***p < 0.001, ****p < 0.0001.

To determine the appropriate therapeutic dose of aspirin, cytotoxicity was tested in HSC‐T6 cells treated with different concentrations of aspirin (0, 5, 10, 15, 20, 25 mmol/L) for 24 hours. As shown in Figure 2A, there was no significant difference in cell viability among the 0 (control), 5, and 10 mmol/L aspirin treatment groups. However, cell viability was significantly reduced at 15, 20, and 25 mmol/L compared to the control group without aspirin treatment (Figure 2A). Therefore, 10 mmol/L of aspirin was used in subsequent experiments.

To further investigate the impact of aspirin on autophagy in vitro, HSCs were treated with 5 ng/mL TGF‐β1 for 24 h, with the addition of ASA at the 12‐hour mark and continued incubation for another 12 hours. Western blot analysis revealed that TGF‐β1 stimulation significantly increased the levels of LC3II/I, Atg5, and Atg7, along with the autophagic substrate p62, indicating impaired autophagic flux. Conversely, aspirin treatment not only reduced HSC‐T6 activation but also reversed the TGF‐β1‐induced upregulation of LC3II/I and p62 (Figure 2B–I). Notably, aspirin did not affect the expression of phosphorylated mTOR (p‐mTOR), suggesting that its modulation of autophagy in HSC‐T6 cells is independent of the mTOR pathway.

As the activation of the autophagic flux leads to a decrease in p62 expression, the accumulation of sequestosome‐1 p62 implies that the degradation of autophagy may be suppressed. TEM analysis revealed a marked increase in phagophores (isolation membranes) and autophagosomes in livers of TAA‐treated mice compared to controls, whereas aspirin administration significantly restored autophagic flux, as evidenced by reduced autophagic intermediates and increased autolysosomes (Figure 2J,K). These ultrastructural data suggest that aspirin exerts its antifibrotic effects by rescuing impaired autophagic degradation.

### 3.3 Inhibition of Autophagy Impaired Aspirin Anti‐Fibrosis Effects In Vitro

**FIGURE 3:**
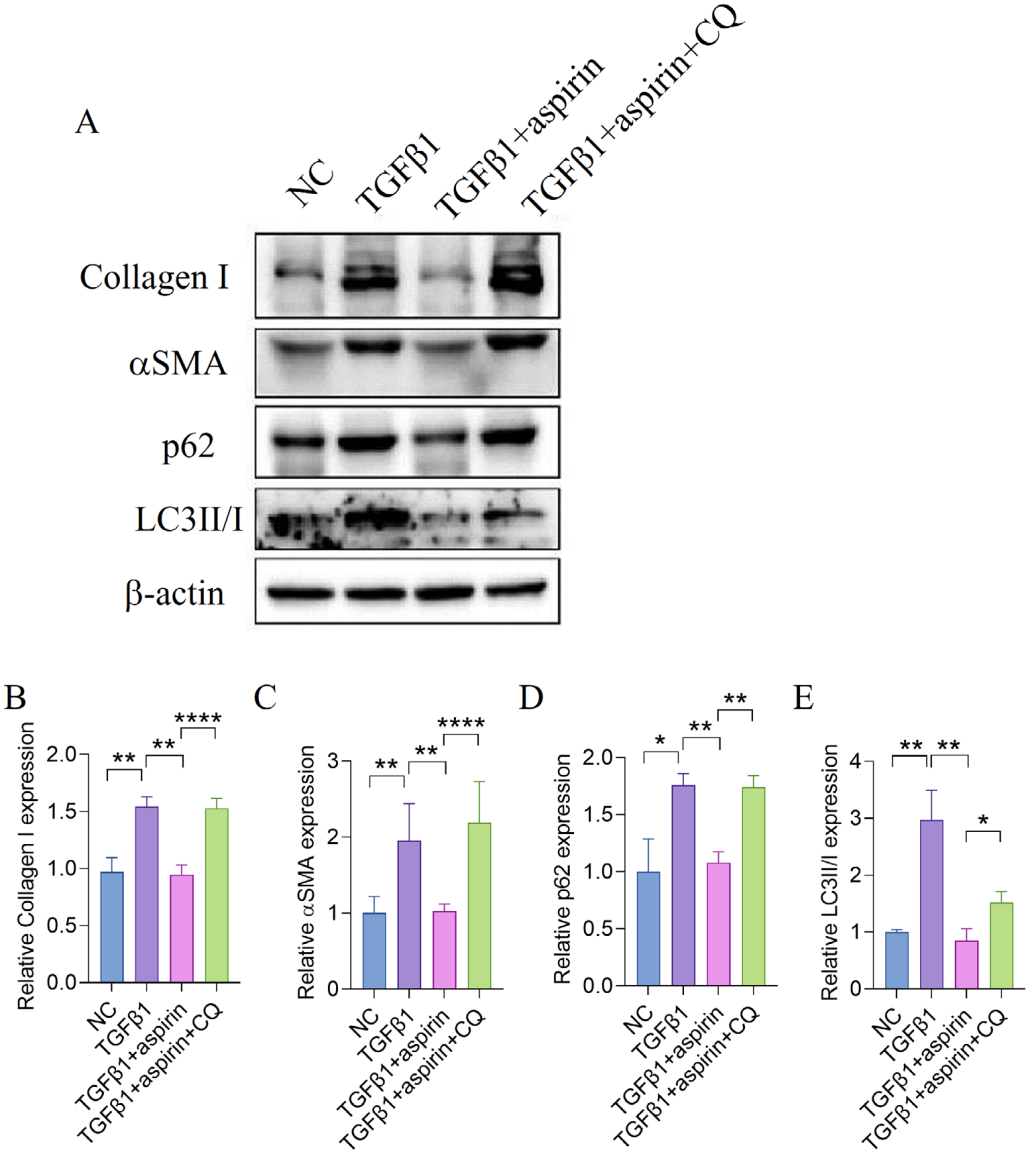
Inhibition of autophagy impaired aspirin's anti‐fibrosis effects in vitro. (A) Analysis of autophagy‐related and fibrosis‐related protein expression in samples from HSC‐T6 cells treated with the indicated treatment. (B–E) Quantification of the indicated protein expression normalised to β‐Actin. For multicomparisons, analysis of variance (ANOVA) was performed to detect an overall difference among the groups. *p < 0.05, **p < 0.01, ***p < 0.001, ****p < 0.0001.

These results indicate that TGF‐β1 treatment inhibits autophagic flux at the terminal stage in HSC‐T6 cells, a phenomenon reversed by aspirin, which promotes autophagosome‐lysosome fusion. To further investigate the role of autophagy in antifibrotic effects of aspirin, we used CQ, a lysosomal inhibitor that blocks autophagosome‐lysosome fusion, to suppress autophagy in activated HSCs. Western blot analysis revealed that CQ treatment abolished the ability of aspirin to enhance autophagic flux, as evidenced by increased levels of LC3‐II/I and p62. These findings confirm that the antifibrotic activity of aspirin is mediated through the promotion of autophagic degradation (Figure 3A–E).

### 3.4 Aspirin Regulates Autophagic Flux Through a Dual‐Mechanism Framework

**FIGURE 4:**
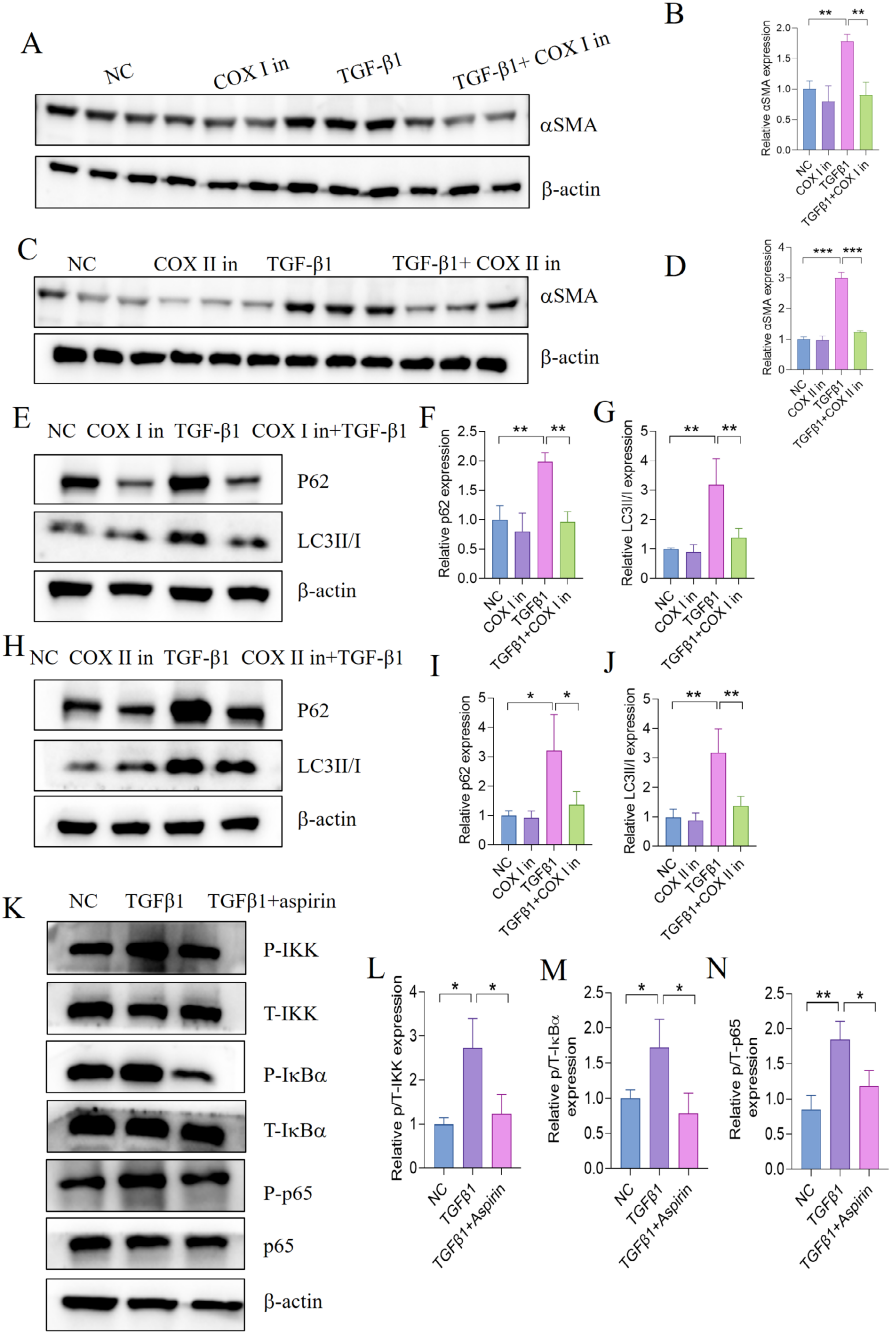
Aspirin regulates autophagic flux through a dual‐mechanism framework. (A, B) HSC‐T6 cells were treated with COX I (50 μM) inhibitor with or without TGFβ1 for 24 hours. Expression levels of αSMA by Western blot analysis. (C–D) HSC‐T6 cells were treated with COX II (50 μM) inhibitor with or without TGFβ1 for 24 hours. Expression levels of αSMA by Western blot analysis. (E–G) HSC‐T6 cells were treated with COX I (50 μM) inhibitor (COX I in) with or without TGFβ1 for 24 hours. Expression levels of LC3II/I and p62 by Western blot analysis. (H–J) HSC‐T6 cells were treated with COX II (50 μM) inhibitor (COX II in) with or without TGFβ1 for 24 hours. Expression levels of LC3II/I and p62 by Western blot analysis. (K–N) HSC‐T6 cells were treated with TGFβ1 with/without aspirin. Expression levels of the NF‐κB pathway by Western blot. Statistical comparisons with the TGFβ group were performed by an unpaired two‐sided t test. *p < 0.05, **p < 0.01, ***p < 0.001, ****p < 0.0001.

To dissect the COX‐dependent mechanism, HSCs were treated with selective COX‐I (SC‐560, 50 μM) (Figure 4A,B) or COX‐II (celecoxib, 50 μM) (Figure 4C,D) inhibitors. Western blot analysis revealed that both inhibitors significantly suppressed HSCs activation (reduced α‐SMA expression) and decreased autophagic markers LC3‐II and p62, mirroring the effects of aspirin (Figure 4E–J). These results suggest that aspirin promotes autophagic flux and inhibits HSCs activation via concurrent COX‐I/II inhibition.

Additionally, to explore non‐COX‐dependent pathways, aspirin‐treated HSCs were analysed for NF‐κB signalling (Figure 4K–N). Aspirin significantly reduced phosphorylation of p65 (p‐p65), IκB kinase (p‐IKK), and IκBα (p‐IκBα), indicating suppression of the NF‐κB pathway. This dual inhibition of COX enzymes and NF‐κB signalling likely converges to enhance autophagic clearance and attenuate fibrotic responses in HSCs.

## 4 Discussion

The present study demonstrates that aspirin alleviates hepatic fibrosis in both activated HSCs and a murine model of liver fibrosis, primarily through modulating autophagic flux via dual COX‐dependent and non‐COX‐dependent mechanisms. These findings provide mechanistic insights into aspirin's antifibrotic effects and highlight its potential as a repurposed therapeutic for fibrotic liver diseases.

In the context of fibroblast or HSCs activation, autophagy plays a pivotal role. It can suppress the transformation of fibroblasts into myofibroblasts by degrading key profibrotic signalling components. For example, autophagy can target TGF‐β1 receptors, such as TβRII. Degradation of TβRII limits the activation of the TGF‐β1/Smad3 pathway, which is a central driver of fibrosis [24]. The crosstalk between inflammation and fibrosis is also modulated by autophagy. In macrophages, autophagy influences their polarisation. Defective autophagy in macrophages enhances NLRP3 inflammasome activation, leading to a shift toward the pro‐inflammatory M1 phenotype and increased release of fibrogenic cytokines like IL‐1β and TNF‐α [25]. In the liver, Kupffer cells rely on autophagy, specifically mitophagy, to clear damaged mitochondria. This process reduces the production of reactive oxygen species (ROS) and TNF‐α secretion, thereby dampening HSCs activation [26]. Metabolic regulation by autophagy is another important aspect. In hepatocytes, autophagy controls lipid droplet turnover. In non‐alcoholic fatty liver disease (NAFLD), Rubicon blocks autophagic progression by inhibiting the autophagosome‐lysosome fusion step. This effect is independent of the mTOR‐dependent autophagic induction pathway, promoting lipid droplet accumulation in hepatocytes and thereby facilitating the development of liver fibrosis [27]. Additionally, impaired mitophagy in HSCs results in overproduction of ROS. ROS exacerbates fibrosis through activation of the NF‐κB and TGF‐β1 signalling pathways [26].

Studies have shown that p62 is a selective substrate for autophagy, meaning that activation of autophagic flux leads to a decrease in p62 expression. On the other hand, accumulation of p62 indicates weakened autophagic flux [28]. Additionally, inhibition of autophagosome‐lysosome fusion may hinder autophagic maturation [28]. Under normal conditions, LC3‐II is evenly distributed in the cytoplasm. Therefore, the accumulation of LC3‐II and autophagosomes may be a result of blocked autophagic flux. Consequently, the observed p62 accumulation and LC3‐II punctate aggregation in activated HSCs reflect inhibition of autophagosome degradation. We observed that autophagic flux was impaired in HSCs upon addition of TGF‐β1. Aspirin significantly suppresses the expression of p62 and LC3‐II/LC3‐I, suggesting that aspirin acts on the terminal stage of autophagy to promote autophagic flux and inhibit liver fibrosis.

The reduction in hepatic fibrosis markers (α‐SMA and Collagen) in aspirin‐treated HSCs and mice directly correlates with enhanced autophagic flux. Autophagy, a critical homeostatic process, is dysregulated in fibrotic conditions, often manifesting as impaired autophagosome degradation and accumulation of dysfunctional components in activated HSCs [28]. Our data show that aspirin reverses this dysfunction: by decreasing p62 accumulation and LC3‐II aggregation, aspirin likely promotes the terminal stages of autophagy (e.g., autophagosome‐lysosome fusion or acidification), thereby clearing pro‐fibrotic cellular debris and inhibiting HSCs activation. This aligns with prior studies linking autophagy enhancement to reduced ECM deposition in fibrosis [10].

Some studies demonstrated that targets of autophagy can improve the inflammatory microenvironment of liver fibrosis [29]. Aspirin, a nonsteroidal anti‐inflammatory drug, is used clinically as an antipyretic, analgesic, and anti‐inflammatory medicine. Our study proposes a dual pathway framework for aspirin‐induced autophagy, integrating both COX‐dependent and non‐COX‐dependent mechanisms. COX‐dependent mechanisms: While prior studies have shown that aspirin may inhibit the COX‐2/PGE2 axis to downregulate mTORC1 and activate autophagy [30], our novel findings uniquely demonstrate that aspirin exerts autophagic effects in HSCs by inhibiting both COX I and COX II, thereby disrupting pro‐fibrotic signalling via this canonical pathway. However, unlike classical mTOR‐dependent autophagy inducers, aspirin did not alter p‐mTOR Ser2448 phosphorylation in TGF‐β1‐stimulated HSCs, demonstrating that its effects on autophagy and HSCs activation are mTOR‐independent. Non‐COX‐dependent mechanisms: Building on literature identifying NF‐κB as a key target of aspirin in diverse disease contexts [31], mechanistic experiments reveal that aspirin suppresses NF‐κB activation in TGF‐β1‐activated HSCs. This inhibition likely converges with autophagic pathways to attenuate pro‐fibrotic markers (α‐SMA) by disrupting the crosstalk between inflammatory and fibrogenic signalling. Together, these dual mechanisms highlight aspirin's multifaceted role in modulating autophagy to counteract hepatic fibrogenesis. The dual‐action mechanism of aspirin‐targeting both COX enzymes and NF‐κB‐offers a therapeutic advantage over single‐pathway inhibitors, as it addresses multiple drivers of fibrosis. Given aspirin's favourable safety profile and low cost, these findings warrant clinical translation, particularly for patients with comorbid conditions where autophagy dysfunction is prevalent.

In conclusion, our study establishes a mechanistic framework for aspirin's antifibrotic effects, linking COX inhibition and NF‐κB suppression to autophagic flux. These findings not only deepen our understanding of liver fibrosis pathogenesis but also propose aspirin as a promising candidate for repurposed antifibrotic therapy.

## Author Contributions

**Shenglan Wang:** conceptualization (equal), funding acquisition (equal), methodology (equal), project administration (equal), resources (equal), writing – original draft (equal). **Min Tang:** data curation (equal), formal analysis (equal), methodology (equal), validation (equal), writing – original draft (equal), writing – review and editing (equal). **Juan Shan:** writing – review and editing (equal). **Changqing Yang:** conceptualization (equal), funding acquisition (equal), supervision (lead). **Mengxue Sun:** writing – review and editing (equal).

## Disclosure

The authors have nothing to report.

## Ethics Statement

All animal studies were conducted in accordance with the Animal Use Protocol and were approved by Shanghai Tongji Hospital (2023‐DW‐0801‐1231).

## Conflicts of Interest

The authors declare no conflicts of interest.

## Supporting information


**Table S1.** Primer sequences used in this study.
**Table S2.** Primary antibodies used in this study.

## Data Availability

The data supporting the findings of this study are available from the corresponding author upon reasonable request.
