# Supplementary material for: Aspirin Attenuates Liver Fibrosis via Autophagy Induction
Source: J Cell Mol Med. 2025 Jul 3;29(13):e70696. doi: 10.1111/jcmm.70696 (PMC12226408; doi:10.1111/jcmm.70696)
Supplement: Supplementary file 1 — Table S1. Primer sequences used in this study. Table S2. Primary antibodies used in this study. [file JCMM-29-e70696-s001.docx]

**Supplemental Tables**

Table S1. Primer sequences used in this study.

| **Primers for Real-time PCR** | |
| --- | --- |
| **protein** | **Sequence (5’→3’)** |
| Rat GAPDH forward | 5’-AGGTCGGTGTGAACGGATTTG -3’ |
| Rat GAPDH reverse | 5’-TGTAGACCATGTAGTTGAGGTCA -3’ |
| Rat αSMA forward | 5’-CCCAGACATCAGGGAGTAATGG -3’ |
| Rat αSMA reverse | 5’-TCTATCGGATACTTCAGCGTCA-3’ |
| Rat TIMP1 forward | 5’-CGAGACCACCTTATACCAGCG-3’ |
| Rat TIMP1 reverse | 5’-ATGACTGGGGTGTAGGCGTA-3’ |
| Rat HMGB1 forward | 5’-GCTGACAAGGCTCGTTATGAA-3’ |
| Rat HMGB1 reverse | 5’-CCTTTGATTTTGGGGCGGTA-3’ |
| Rat Col1a1 forward | 5’-TAAGGGTCCCCAATGGTGAGA-3’ |
| Rat Col1a1 reverse | 5’-GGGTCCCTCGACTCCTACAT-3’ |

Table S2. Primary antibodies used in this study.

| **Antibodies used in the research** | | |
| --- | --- | --- |
| **Protein** | **Usage** | **Antibody** |
| T-mTOR | WB | T55306 |
| P-mTOR | WB | [PC3495](http://www.ab-mart.com.cn/page.aspx?node= 77 &id= 287255" \t "https://www.ab-mart.com.cn/_blank) |
| Atg5 | WB | [T55766](http://www.ab-mart.com.cn/page.aspx?node= 77 &id= 2004" \t "https://www.ab-mart.com.cn/_blank) |
| Atg7 | WB | T57051 |
| P-IKK | WB | TP56290 Abmart |
| T-IKK | WB | T55735 Abmart |
| P-IκBα | WB | TC60711S Abmart |
| T-IκBα | WB | T55026 Abmart |
| P62 | WB | 3087S Cell signaling technology |
| LC3 | WB | #12741 Cell signaling technology |
| P62 | WB | 66184-1-Ig Proteintech |
| P65 | WB | [TA5006](http://www.ab-mart.com.cn/page.aspx?node= 77 &id= 20069" \t "https://www.ab-mart.com.cn/_blank) Abmart |
| P-P65 | WB | Tp56372 Abmart |
| αSMA | WB | Ab32575 Abmart |
| Beclin | WB | [T55092](http://www.ab-mart.com.cn/page.aspx?node= 77 &id= 1392" \t "https://www.ab-mart.com.cn/_blank) Abmart |

Abbreviations: WB, Western blotting
